# Supplementary material for: MET Oncogene Enhances Pro-Migratory Functions by Counteracting NMDAR2B Cleavage
Source: Cells. 2023 Dec 21;13(1):28. doi: 10.3390/cells13010028 (PMC10777984; doi:10.3390/cells13010028)
Supplement: Supplementary file 1 [file cells-13-00028-s001.zip › cells-2760673-supplementary.pdf]

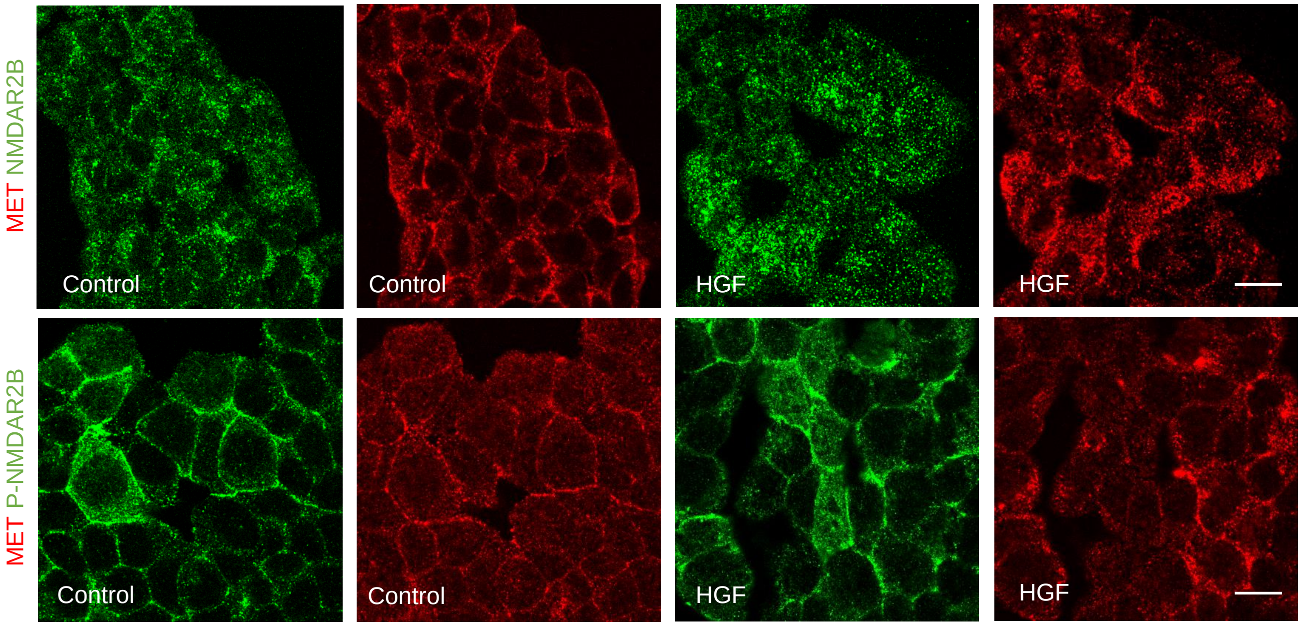

Supplementary Figure S1. Single-channel images for MET (red) and total (upper panels) or phosphorylated (Tyr1252, lower panels) NMDAR2B (green) proteins from the merged confocal double immunofluorescence shown in Figure 1a. Bar = 50 μm.

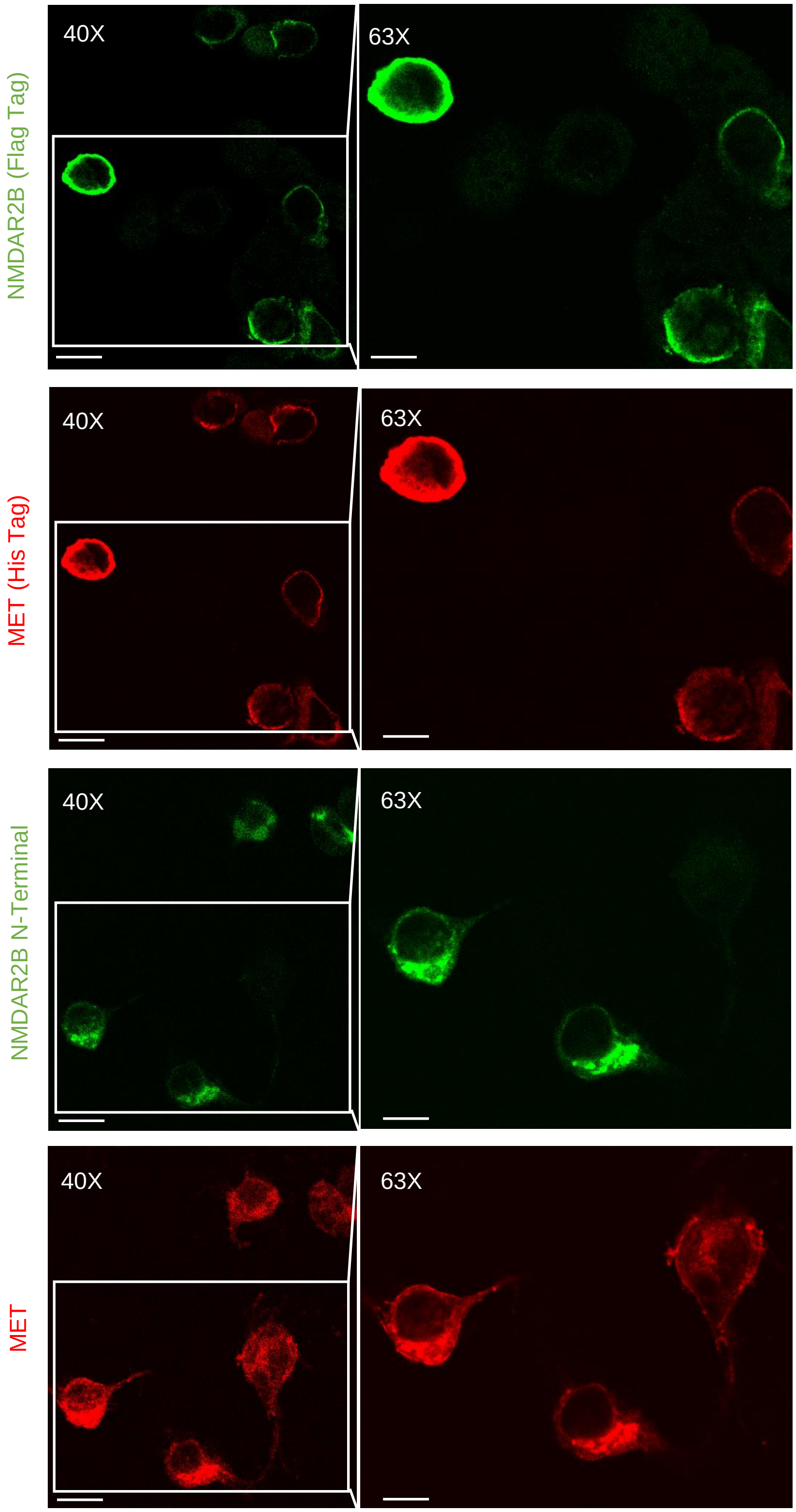

Supplementary Figure S2. Single-channel images from the merged confocal double immunofluorescence shown in Figure 2c. Anti-Flag (NMDAR2B, green); anti-His (MET, red); anti-N-terminal NMDAR2B (green); anti-MET (red) antibodies. Images were taken by two enlargements (40 and 63X). Bar 40X = 50 μm, Bar 63X = 80 μm.

Coordinate	Analyte	Entrex Gene ID	Coordinate	Analyte	Entrex Gene ID
A1, A2	Reference Spots	N/A	E1, E2	CXCL8/IL-8	3576
A3, A4	α-Fetoprotein	174	E3, E4	IL-18 BPa	10068
A5, A6	Amphiregulin	374	E5, E6	Kallikrein 3/PSA	354
A7, A8	Angiopoietin-1	284	E7, E8	Kallikrein 5	25818
A9, A10	Angiopoietin-like 4	51129	E9, E10	Kallikrein 6	5653
A11, A12	ENPP-2/Autotaxin	5168	E11, E12	Leptin	3952
A13, A14	Axl	558	E13, E14	Lumican	4060
A15, A16	BCL-x	598	E15, E16	CCL2/MCP-1	6347
A17, A18	CA125/MUC16	94025	E17, E18	CCL8/MCP-2	6355
A19, A20	E-Cadherin	999	E19, E20	CCL7/MCP-3	6354
A21, A22	VE-Cadherin	1003	E21, E22	M-CSF	1435
A23, A24	Reference Spots	N/A	E23, E24	Mesothelin	10232
B3, B4	CapG	822	F1, F2	CCL3/MIP-1α	6348/6351
B5, B6	Carbonic Anhydrase IX	768	F3, F4	CCL20/MIP-3α	6364
B7, B8	Cathepsin B	1508	F5, F6	MMP-2	4313
B9, B10	Cathepsin D	1509	F7, F8	MMP-3	4314
B11, B12	Cathepsin S	1520	F9, F10	MMP-9	4318
B13, B14	CEACAM-5	1048	F11, F12	MSP/MST1	4485
B15, B16	Decorin	1634	F13, F14	MUC-1	4582
B17, B18	Dkk-1	22943	F15, F16	Nectin-4	81607
B19, B20	DLL1	28514	F17, F18	Osteopontin	6696
B21, B22	EGF R/ErbB1	1956	F19, F20	p27/Kip1	1027
C3, C4	Endoglin/CD105	2022	F21, F22	p53	7157
C5, C6	Endostatin	80781	F23, F24	PDGF-AA	5154
C7, C8	Enolase 2	2026	G1, G2	CD31/PECAM-1	5175
C9, C10	eNOS	4846	G3, G4	Progesterone R/NR3C3	5241
C11, C12	EpCAM/TROP1	4072	G5, G6	Progranulin	2896
C13, C14	Erα/NR3A1	2099	G7, G8	Prolactin	5617
C15, C16	ErbB2	2064	G9, G10	Prostasin/Prss8	5652
C17, C18	ErbB3/Her3	2065	G11, G12	E-Selectin/CD62E	6401
C19, C20	ErbB4	2066	G13, G14	Serpin B5/Maspin	5268
C21, C22	FGF basic	2247	G15, G16	Serpin E1/PAI-1	5054
D1, D2	FoxC2	2303	G17, G18	Snail	6615
D3, D4	FoxO1/FKHR	2308	G19, G20	SPARC	6678
D5, D6	Galectin-3	3958	G21, G22	Survivin	332
D7, D8	GM-CSF	1437	G23, G24	Tenascin C	3371
D9, D10	CG α/β (HCG)	1081 (α)/1082 (β)	H1, H2	Thrombospondin-1	7057
D11, D12	HGF R/c-Met	4233	H3, H4	Tie-2	7010
D13, D14	HIF-1α	3091	H5, H6	u-Plasminogen Activator/Urokinase	5328
D15, D16	HNF-3β	3170	H7, H8	VCAM-1/CD106	7412
D17, D18	HO-1/HMOX1	3162	H9, H10	VEGF	7422
D19, D20	ICAM-1/CD54	3383	H11, H12	Vimentin	7431
D21, D22	IL-2 Rα	3559	I1, I2	Reference Spots	N/A
D23, D24	IL-6	3569	I23, I24	Negative Control	N/A

Table S1. Scheme of the human oncology proteins antibody arrays used in the work. Human XL Oncology Array allow to analyze 84 cancer-related proteins.

Primary Antibody	Company	Product N.	Experiment
NMDAR2B C-Terminal	Abcam	ab65783	WB/IF
P-NMDAR2B (Tyr1252)	Invitrogen	48-5200	WB/IF
MET	R&D	AF276	IF
MET	Cell Signaling	D1C2 - 8198	WB
P-MET (Tyr1234/1235)	Cell Signaling	3077	WB
Tubulin	SigmaAldrich	051M4771	WB
NMDAR2B N-Terminal	Abcam	ab93610	WB
His Tag	R&D	mab050	IF
Flag Tag	SigmaAldrich	F7425	IF
LC3B	SigmaAldrich	L7543	WB
p70S6K	Cell Signaling	9202	WB
P-p70S6K (Thr389)	Cell Signaling	9205	WB

Table S2. List of primary antibodies used throughout the study.
